# Molecular Characterization and Functional Analysis of Two Petunia *PhEILs*

**DOI:** 10.3389/fpls.2016.01606

**Published:** 2016-11-01

**Authors:** Feng Liu, Li Hu, Yuanping Cai, Hong Lin, Juanxu Liu, Yixun Yu

**Affiliations:** ^1^Guangdong Key Laboratory for Innovative Development and Utilization of Forest Plant Germplasm, College of Forestry and Landscape Architecture, South China Agricultural UniversityGuangzhou, China; ^2^College of Horticulture, South China Agricultural UniversityGuangzhou, China

**Keywords:** petunia, VIGS, flower senescence, ethylene signaling, EIL

## Abstract

Ethylene plays an important role in flower senescence of many plants. *Arabidopsis* ETHYLENE INSENSITIVE3 (EIN3) and its homolog EIL1 are the downstream component of ethylene signaling transduction. However, the function of EILs during flower senescence remains unknown. Here, a petunia *EIL* gene, *PhEIL2*, was isolated. Phylogenetic tree showed that PhEIL1, whose coding gene is previously isolated, and PhEIL2 are the homologs of *Arabidopsis* AtEIL3 and AtEIL1, respectively. The expression of both *PhEIL1* and *PhEIL2* is the highest in corollas and increased during corolla senescence. Ethylene treatment increased the mRNA level of *PhEIL1* but reduced that of *PhEIL2*. VIGS-mediated both *PhEIL1* and *PhEIL2* silencing delayed flower senescence, and significantly reduced ethylene production and the expression of *PhERF3* and *PhCP2*, two senescence-associated genes in petunia flowers. The PhEIL2 protein activating transcription domain is identified in the 353-612-amino acids at C-terminal of PhEIL2 and yeast two-hybrid and bimolecular fluorescence complementation assays show that PhEIL2 interacts with PhEIL1, suggesting that PhEIL1 and PhEIL2 might form heterodimers to recognize their targets. These molecular characterizations of PhEIL1 and PhEIL2 in petunia are different with those of in *Vigna radiata* and *Arabidopsis*.

## Introduction

Plant hormone ethylene plays an important role in plant growth and development (Abeles et al., 1992). Ethylene perception is mediated by ETR1 family (Chang et al., 1993; Hua et al., 1995, 1998; Schaller and Bleecker, 1995). CONSTITUTIVE TRIPLE RESPONSE 1 (CTR1), a Raf-like Ser/Thr protein kinase, functions in downstream of receptors and in upstream of the central regulator ETHYLENE-INSENSITIVE2 (EIN2) (Kieber et al., 1993; Alonso et al., 1999). EIN2 is phosphorylated by CTR1 to trigger its endoplasmic reticulum (ER)–to–nucleus translocation and to control ethylene signaling from the ER membrane to the nucleus (Ju et al., 2012; Qiao et al., 2012). The C-terminal end of EIN2 (CEND) is thought to participate in signaling output, as ectopic expression of this domain alone can partially activate ethylene responses (Alonso et al., 2003; Wen et al., 2012). The CEND of EIN2 can be phosphorylated by the receptors-activated CTR1 in the absence of ethylene and phosphorylation-regulated proteolytic processing of EIN2 triggers its ER-to-nucleus translocation (Ju et al., 2012; Qiao et al., 2012). Inhibition of CTR1 upon ethylene perception is a signal for cleavage and nuclear localization of the EIN2 C terminus to stabilize EIN3 protein (Wen et al., 2012; Ji and Guo, 2013).

In *Arabidopsis*, EIN3 and three related EIN3-LIKE (EIL1, EIL2, and EIL3) proteins were shown to possess amino acid sequence similarity and conserved structural features for nuclear-localized transcription factors (Chao et al., 1997). The ethylene signal is transmitted to the EIN3 family of transcription factors, which have been shown to act as a transcriptional activator and bind to the primary ethylene-response element present in the promoter of the ethylene-responsive *ERF1* gene (Chao et al., 1997; Solano et al., 1998). One found that the control of EIN3 degradation is important to regulation of ethylene signaling transduction (Yanagisawa et al., 2003). Two F-box proteins, EBF1 and EBF2 interact with EIN3 and EIL1 (Guo and Ecker, 2003; Potuschak et al., 2003), and disruption of either *EBF1* or *EBF2* leads to the increase of EIN3 protein levels and induces a hypersensitivity to ethylene. The *ebf1 ebf2* double mutant results in a large accumulation of EIN3 proteins and causes a constitutive ethylene response phenotype (Gagne et al., 2004).

Recently, another mechanism of EIN2-mediated ethylene signaling was reported in *Arabidopsis* (Li et al., 2015). The translational repression of *EBF1* and *EBF2* transcription is imposed by EIN2. The EIN2-directed translational repression is mediated by the *EBF1/2* 3′UTRs and multiple poly-uridylates (PolyU) motifs are identified as functional *cis* elements of 3′UTRs (Li et al., 2015).

Ethylene production is increased during flower senescence in many flowers, including petunia, often as the model system for studying the biological bases of flower senescence (Borochov and Woodson, 1989). However, the function of petunia EILs during flower senescence is not well known.

In petunia *EIL* family, *PhEIL1* (accession no. Y353248) has been identified and its expression in response ethylene treatment was reported (Shibuya et al., 2004). Here, another full-length cDNA of petunia *EIL* gene, *PhEIL2*, was cloned. *PhEIL1* and *PhEIL2* expression profile was established in different petunia tissues, at various stages of flower senescence and in response to ethylene. VIGS-mediated both *PhEIL1* and *PhEIL2* silencing delayed flower senescence in petunia and reduced the expression of two senescence-associated genes. Yeast two-hybrid (Y2H) and bimolecular fluorescence complementation (BiFC) assays showed that PhEIL2 interacts with PhEIL1.

## Materials and Methods

### Plant Material

Petunia (*Petunia hybrida* ‘Ultra’) plants were grown under normal greenhouse conditions (22°C, 14-h light/10-h dark). Flowers were emasculated 1 day before flowers were fully open to prevent self-pollination. Eight to ten petunia flowers were harvested at anthesis stages and placed in distilled water for further processing. Corollas were collected from petunias at 0, 1, 2, 3, 4, 5, 6, 7, and 8 days after flower opening. Stems, leaves and roots were collected from plants at the vegetative stage when the plants were about 10 cm in height. These tissues were firstly frozen in liquid nitrogen and then stored at -80°C. All experiments were performed at least three times.

### RNA Extraction and RT-PCR

RNA extraction and RT-PCR was performed according to the previous protocols (Liu et al., 2010). The RNA content was determined spectrophotometrically. One microgram of total RNA was reverse transcribed at 42°C for 1 h in a final volume of 20 μl containing reaction buffer, 20 mmol l^-1^ DTT, 0.5 mmol l^-1^ dNTP, 1 μg Oligo (dT) 15 and reverse transcriptase (AMV, Promega, USA) according to the manufacturer’s instructions.

### Cloning of the Petunia *PhEIL2* Gene

The partial sequence of *PhEIL2* was obtained by to the previously described approach (Yang et al., 2009). In brief, TBLASTN analysis against the Genebank EST database^1^ with *AtEIN3* and *AtEIL1* identified one petunia clone, FN029455, which encodes putative protein displaying high homology with AtEIN3 and AtEIL1, respectively.

The remaining 5′ and 3′ cDNA sequences of *PhEIL2* were cloned by rapid-amplification of cDNA ends (RACE) with the forward primer 5′CTATCCTGATCGCTGCCCACCT3′ and revered primer 5′TCTCCAATGGAAATCTTCTCTG3′ (Liu et al., 2010).

### Sequence Analysis

The neighbor-joining tree at amino acid level was drawn by DNAMAN software. The reliability of each branch of the tree was assessed using 1,000 bootstrap replications. Identity search for nucleotides and translated amino acids was carried out using National Center for Biotechnology Information (NCBI) BLAST network server^2^.

### Ethylene Measurements

Petunia flowers were treated with ethylene according to previously described protocols (Tan et al., 2014). To measure ethylene production, corollas of each individual flower were collected and placed in a 200 ml airtight container according to the method of Liu et al. (2010). Thus, to avoid the contamination of wound-induced ethylene, the containers were capped and incubated at 25°C for 1 h. Next, a 2 ml sample of head-space gas was withdrawn using a gas-tight hypodermic syringe and was injected into a gas chromatograph (GC 17A, Shimadzu, Kyoto, Japan) to measure the ethylene concentration. The gas chromatograph was equipped with a flame ionization detector and an activated alumina column. All measurements were performed in five replicates.

### Quantitative Real-Time PCR Assays

Total RNA extracted from various tissues was digested with DNAase I and then reverse transcription (RT) was performed according to the kit instruction (TaKaRa, China). PCR analysis was carried out with the cDNA as a template. Specific primer design was performed using the sequences obtained for *PhEIL1, PhEIL2, PhERF3*, and *PhCP2*. The petunia *Actin* (accession no. FN014209) genes were used as the internal reference gene to quantify the cDNA abundance (Mallona et al., 2010). The sequences of all primers used for qPCR analysis are described in Supplementary Table S2.

### Ethylene and Pollination Treatment

Petunia flowers were treated with ethylene according to the previously described protocols (Yang et al., 2015). Petunia flowers were harvested at anthesis and their stems re-cut to 5 cm, placed in flasks with distilled water, and subsequently treated with 2 μl l^-1^ ethylene for 0, 2, 4, 8, 12, and 24 h. To measure the expression of *PhEIL1* and *PhEIL2* after pollination, three flowers from each of six plants (18 flowers in total per genotype) for different lines were self-pollinated on the plant on the day before anthesis (Shibuya et al., 2004). Corollas from 8 to 10 flowers were collected at each time point, immediately frozen in liquid nitrogen, and stored at -80°C for later RNA extraction.

### Agroinoculation of TRV Vectors

Approximately 250 bp 3′ untranslated regions of *PhEIL1* and *PhEIL2* were cloned into the pTRV2-CHS vector to formed TRV2-CHS-PhEIL1 and TRV2-CHS-PhEIL2 vectors using their respective forward and reverse primers (Supplementary Table S3). pTRV1 and different pTRV2 derivatives vectors were transformed into *Agrobacterium tumefaciens* (strain GV3101) (Spitzer-Rimon et al., 2010). The *Agrobacterium* culture was grown overnight at 28°C in Luria-Bertani medium with 50 mg l^-1^ kanamycin and 200 mM acetosyringone. The cells were harvested and resuspended in inoculation buffer containing 10 mM MES, pH 5.5, 200 mM acetosyringone, and 10 mM MgCl_2_ to an OD_600_ of 10. Following an additional 3 h of incubation at 28°C, the bacteria containing pTRV1 were mixed with the bacteria containing the pTRV2 derivatives in a 1:1 ratio. Next, 200 to 300 ml of this mixture was applied to the cut surface of petunia plantlets after the removal of the apical meristems.

### Flower Longevity

Flower longevity was measured according to previously described methods (Tan et al., 2014). To measure flower senescence, three flowers were removed from each of 20 plants (60 flowers in total per genotype) from the wild type (purple) and VIGS-mediated gene suppression lines (white) on the day before anthesis, and the flowers were placed in vials of water. The flowers were then placed in a growth room under continuous fluorescent light at 24–26°C, and the day on which each flower completely wilted was recorded.

The data were analyzed using the ANOVA function of SAS 8.02 (Cary, NC, USA) to compare differences among genotypes. Tukey’s honestly significant difference mean-separation test was used to calculate the mean separation at the 0.05% level (HSD0.05).

### Deletion Mutant Construction of PhEIL2 and Analyze of Transactivation Activity

The deletion mutants of PhEIL2 were constructed by PCR. The sequences of all primers used for various PhEIL2 deletion mutants are described in Supplementary Table S4. The PCR products were fused in frame to the yeast GAL4 DNA-binding domain expression vector pGBKT7. The constructed vectors were transformed into *Saccharomyces cerevisiae* strain Y2HGold (Clontech, Palo Alto, CA, USA). The β-galactosidase assay was performed according to the kit instructions (Clontech).

### Yeast Two-Hybrid Analysis

The coding sequence of PhEIL2 was cloned into the bait vector pGBKT7, and the coding sequences of *PhEIL1* were cloned into the prey vector pGADT7. The gene-specific primers of the three genes are shown in Supplementary Table S5. The mating reactions were performed between the two haploid strains containing the pGBKT7-PhEIL2 and pGADT7-PhEIL1 constructs and were plated on double dropout medium (DDO medium, SD/–Leu/–Trp) (BD Biosciences Clontech). The transformants were further streaked on quadruple dropout medium (QDO medium, SD/–Trp/–Leu/–His/–Ade) and were confirmed with a color change on β-galactosidase filter paper using a flash-freezing filter assay (Tan et al., 2014).

### Bimolecular Fluorescence Complementation (BiFC) Assay

The full-length *PhEIL1* and *PhEIL2* cDNAs were inserted into pSAT-1628 (pYFC) and pSAT-1882 (pYFN) to form the pYFC-PhEIL1 and pYFN-PhEIL2 vectors, respectively. The sequences of all primers used for BiFC are described in Supplementary Table S6. pYFC-PhEIL1 and pYFN-PhEIL2, pYFC-PhEIL1 and pSAT-YFC, pSAT-YFN, and pYFN-PhEIL2 were co-transformed into petunia protoplasts with the pSAT-GFP as positive control (Tan et al., 2014).

Leaves of 5- to 6-week-old petunia plants were used for the preparation of protoplasts. The vectors were used in polyethylene glycol-mediated transformation of the petunia protoplasts (Spitzer-Rimon et al., 2010). The protoplasts were assayed for fluorescence 12–24 h after transformation. The images were produced by the confocal laser scanning system (ECLIPSE TE2000-E; Nikon, Tokyo, Japan).

### Statistical Analyses

Statistical analysis was performed using one way analysis of variance (ANOVA) followed by Duncan’s multiple range test (DMRT) with three replicates. *P*-values ≤ 0.05 were considered as significant.

## Results

### Identification of a PhEIL Transcription Factor Gene in Petunia

A *PhEIL* transcription factor gene full-length cDNA was isolated in the petunia ‘Ultra,’ named *PhEIL2*, since *PhEIL1* had been previously submitted to GeneBank. *PhEIL2* was predicted to encode a 612 amino acid protein, with a calculated molecular weight of 69.1 kDa. The multiple sequence alignments of EIL-like proteins in petunia, tomato and *Arabidopsis* are presented in Supplementary Figure S1. PhEIL1 shares 35.1, 35.6, 29.7, 50.1, and 34.6% amino acid sequence identity with PhEIL2, AtEIL1, AtEIL2, AtEIL3, and AtEIN3. PhEIL2 shares 62.7, 40.2, 39.3, and 61.8% amino acid sequence identity with AtEIL1, AtEIL2, AtEIL3, and AtEIN3 (Supplementary Table S1). The N-terminal half of the deduced protein of PhEIL1 and PhEIL2 had higher similarity to the corresponding regions of AtEIL3 and AtEIL1 in *Arabidopsis* than their C-terminal half to the corresponding regions of the latter two, respectively (Supplementary Figure S1). As found in other EIN3 homologs, the PhEIL1 and PhEIL2 proteins possess an amino-terminal acidic region, five small clusters of basic amino acids regions and a Pro-rich domain (158–198; 201–242, respectively) (Supplementary Figure S1). The acidic and Pro-rich regions have been proposed to be functional as transcriptional activation domains (Chao et al., 1997). Acidic and proline-rich regions have been widely described as transcriptional activation domains (Mitchell and Tjian, 1989) and may serve such a role in the EIN3/EIL proteins. PhEIL1 and PhEIL2 have a Lys residue at positions 203 and 247, respectively, which is required for the function of EIN3 (Chao et al., 1997; Solano et al., 1998; Alonso et al., 2003). In addition, phylogenetic tree showed that PhEIL1 is the homologs of AtEIL3 and PhEIL2 is the homologs of AtEIL1 and AtEIN3 (Supplementary Figure S2).

### Expression of *PhEIL1* and *PhEIL2*

**FIGURE 1:**
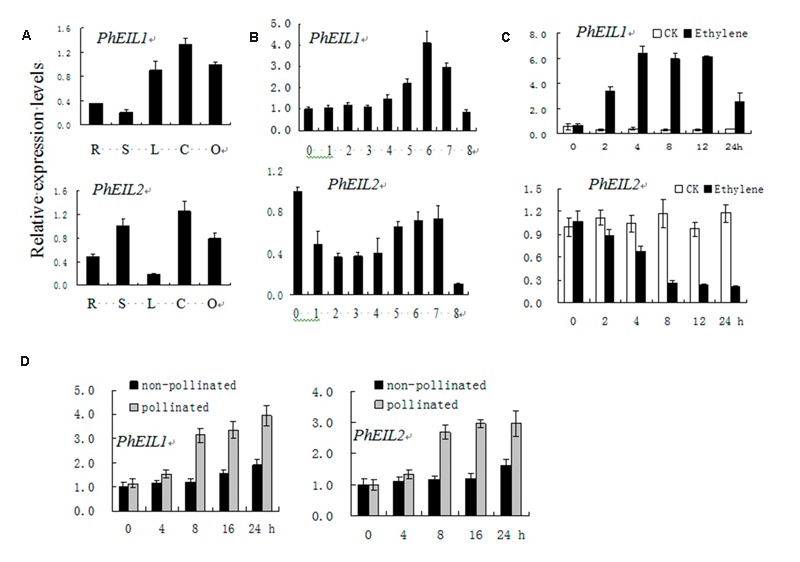
**Temporal and spatial expression analysis of *PhEIL1* and *PhEIL2* by quantitative real-time PCR.** Expression analysis of *PhEIL1* and *PhEIL2* in different organs **(A)**, in corollas during natural flower senescence **(B)**, response to ethylene **(C),** and pollination **(D)**. R, roots; L, leaves; S, stems; C, corollas, O, ovaries. Relative expression levels are shown as fold change values. Data are the mean ± SD (*n* = 3). Statistical analysis was performed using one way analysis of variance (ANOVA) followed by Duncan’s multiple range test (DMRT) with three replicates. *P*-values ≤ 0.05 were considered as significant.

Quantitative real-time PCR (qPCR) analysis showed that both *PhEIL1* and *PhEIL2* mRNA levels are high in corollas and ovaries, and the expression *PhEIL1* is the lowest in stems, while that of *PhEIL2* is the lowest in leaves (**Figure 1A**). During natural flower senescence, the *PhEIL1* mRNA level did not show significant change before day 3 but increased rapidly from days 4 to 6. The expression of *PhEIL2* decreased before day 3, but then increased until day 7 (**Figure 1B**). After ethylene treatment, *PhEIL1* mRNA levels significantly increased from hours 2 to 24, whereas *PhEIL2* expression decreased from 0 to 8 h and then kept stable level until to 24 h (**Figure 1C**). In petunia, pollination induced an ethylene burst and consequently floral senescence (Shibuya et al., 2004). As shown as in **Figure 1D**, the mRNA levels of PhEIL1/PhEIL2 were significantly increased after pollination 8 h by qPCR analysis.

### Silencing of Both *PhEIL1* and *PhEIL2* Delays Flower Senescence and Decrease Ethylene Production

**FIGURE 2:**
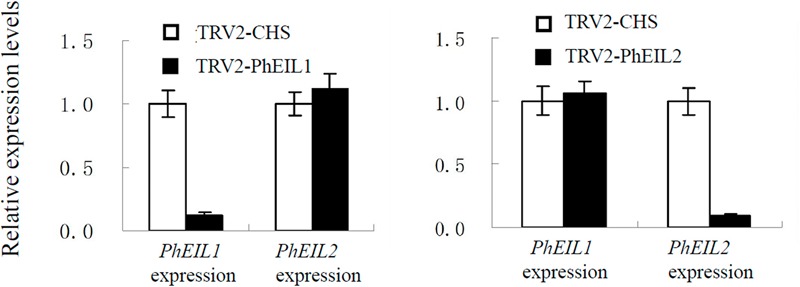
**Effects of TRV2-CHS/PhEIL1 and TRV2-CHS/PhPhEIL2 treatment on the expression of *PhEIL1* and *PhEIL2* in white flowers on day 4 after opening by quantitative real-time PCR, respectively.** Relative expression levels are shown as fold change values. Data are mean ± SD (*n* = 3). Statistical analysis was performed using one way analysis of variance (ANOVA) followed by Duncan’s multiple range test (DMRT) with three replicates. *P*-values ≤ 0.05 were considered as significant.

The VIGS system with *PhCHS* as the reporter gene has been established in the petunia ‘Ultra’ (Violet line) by us (Tan et al., 2014). To identify the effects of *PhEIL1* and *PhEIL2* silencing on flower senescence, TRV-CHS-PhEIL1 and TRV-CHS-PhEIL2 vectors, which were inserted approximately 250 bp fragments of 3′ untranslated sequences of the petunia *PhEIL1* and *PhEIL2* cDNAs into a pTRV2-CHS vector, respectively, were constructed. The *PhEIL1* and *PhEIL2* mRNA levels in the white flowers in the TRV-CHS-PhEIL1 and TRV-CHS-EIL2 treatments, respectively, decreased to less than 20% relative to control (TRV-CHS), and *PhEIL2* and *PhEIL1* mRNA levels did not significantly changed in the flowers of *PhEIL1* and *PhEIL2* silencing, respectively (**Figure 2**).

**FIGURE 3:**
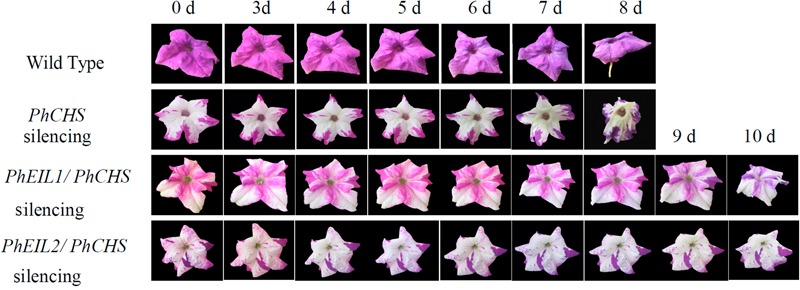
**Effects of *PhCHS* silencing, *PhCHS/PhEIL1* silencing, and *PhCHS/PhEIL2* silencing on the longevity of petunia flowers.** Petunia plants were infected with TRV CHS, TRV PhCHS/PhEIL1, or TRV PhCHS/PhEIL2. Flowers showing the white silencing phenotype and purple flowers from uninfected plants were excised and photographed every day after opening.

As shown in **Figure 3** and **Table 1**, the longevity of the flowers of *PhCHS/PhEIL1* silencing and *PhCHS/PhEIL2* silencing was increased compared with that of the flowers from plants of *PhCHS* silencing and wild type plants.

**Table 1:** The effects of PhEIL1 and PhEIL2 suppression on the longevity of petunia flowers.

	Wild type	PhCHS suppression	PhEIL1 suppression	PhEIL2 suppression
longevity of flowers (day)	6.96 ± 0.82^c^	7.0 ± 0.8^c^	8.4 ± 0.9^b^	10.6 ± 1.3^a^

*Data were expressed as mean ± SD. Different letters indicate significant difference among treatments at the 0.05 significance level based on Duncan’s multiple-range test. *Error bars* indicate standard error of the mean (*n* = 3).*

**FIGURE 4:**
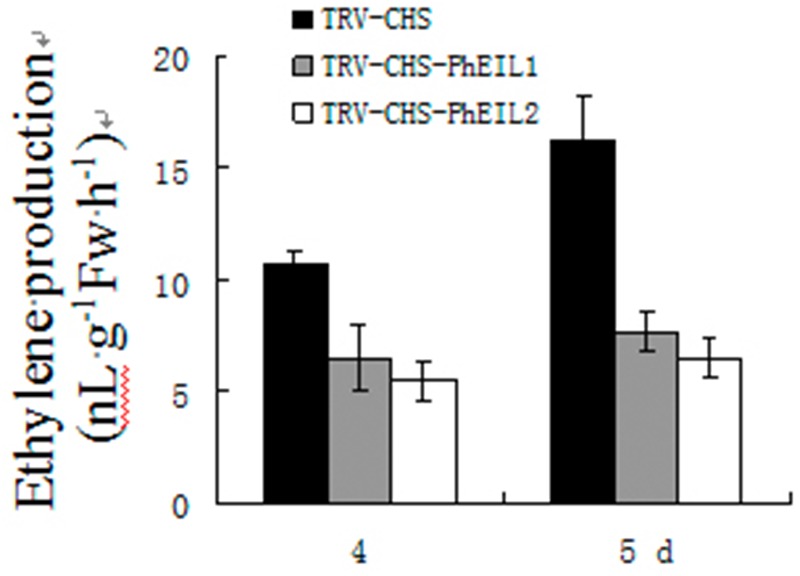
**Ethylene productions in *PhEIL1* and *PhEIL2* silencing flowers.** Ethylene production was measured for flower corollas after the flowers were open for 4 and 5 days. Ethylene was collected for 2 h and subsequently measured using a gas chromatograph. Ethylene production rates were calculated based on tissue FW. Mean ± SE values were determined from five samples. Statistical analysis was performed using one way analysis of variance (ANOVA) followed by Duncan’s multiple range test (DMRT) with three replicates.

The effects of *PhEIL1* and *PhEIL2* silencing on ethylene production were examined. As shown in **Figure 4**, white flowers of both *PhCHS/PhEIL1* and *PhCHS/PhEIL2* silencing produced less ethylene than those of *PhCHS* silencing (TRV-CHS treatment) in days 4 and 5 after anthesis.

### Silencing of Both *PhEIL1* and *PhEIL2* Reduces the Expression of *PhERF3* and *PhCP2*

**FIGURE 5:**
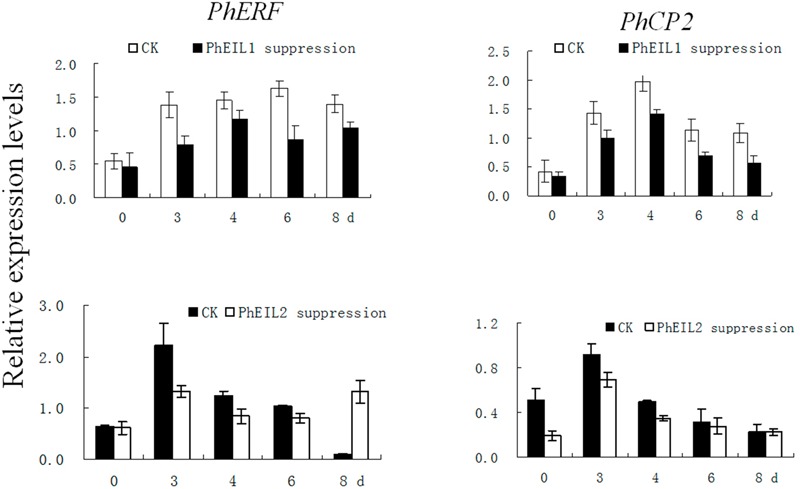
**Effects of *PhEIL1* and *PhEIL2* silencing on the expression of *PhERF3* and *PhCP2* in flowers.** Flowers were detached on the day in anthesis. Total RNA was isolated from white flower tissues. *PhERF3* and *PhCP2* mRNA levels were determined by quantitative real-time PCR. Relative expression levels are shown as fold change values. Data are the mean ± SD (*n* = 3). Statistical analysis was performed using one way analysis of variance (ANOVA) followed by Duncan’s multiple range test (DMRT) with three replicates. *P*-values ≤ 0.05 were considered as significant.

In petunia, *PhERF3* (HQ259597) and *PhCP2* (AY662988) are regard as senescence-associated genes (Jones et al., 2005; Liu et al., 2010). To further examine the involvement of PhEIL1 and PhEIL2 in flower senescence, the expression of *PhERF3* and *PhCP2* was examined by qPCR in *PhEIL1-* and *PhEIL2*-silenced flowers. Expression of *PhERF3* and *PhCP2* in white flowers with *PhCHS/PhEIL1* and *PhCHS/PhEIL2* silencing was significantly lower than that with *PhCHS* silencing after the flowers were open for 4 and 5 days (**Figure 5**).

### The PhEIL2 Protein Activate Transcription in Yeast

**FIGURE 6:**
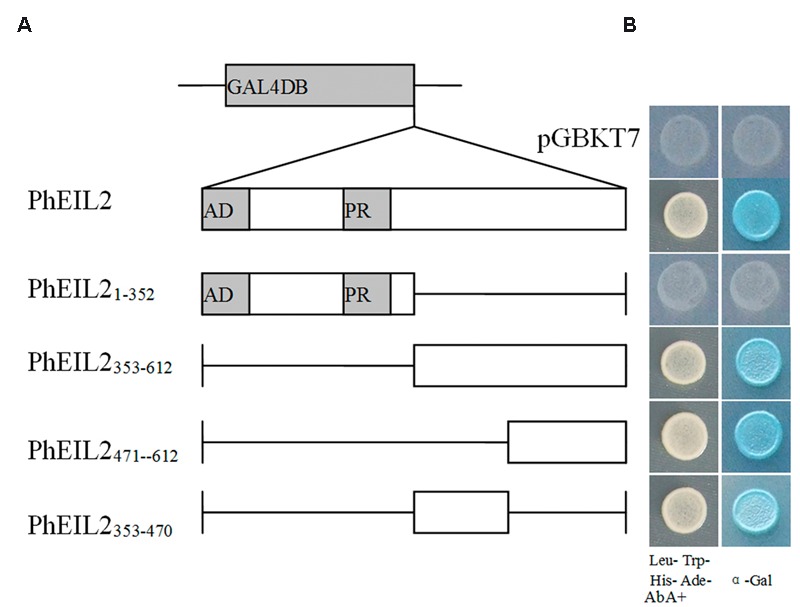
**GAL4 DB-PhEIL2 fusions and their effect on transcriptional activation of the *lacZ* reporter gene in yeast cells. (A)** Schematic overview of the fusion proteins between the GAL4 DNA-binding domain (DB) and various deletion mutants of PhEIL2 that were investigated for transcription-stimulating activity in yeast. **(B)** The *PhEIL2* and its deletion constructs fused in the GAL4 DB expression vector were transformed into yeast strain Y2HGold. The transformants were selected by growth on Trp- and Leu- medium at 30°C for 3 days. Yeast transformants were tested for growth in the absence of His, Trp, Leu, and Ade but containing 125 μM Aureobasidin A as well as turn blue in the presence of the chromagenic substrate X-β-Gal was scored as a positive interaction. The yeast strain is carrying a modified *HIS* gene whose transcription is under the control of GAL4 operator.

Previous research suggested that the N-terminal acidic region (1–50 amino acids) of mung bean (*Vigna radiata*) VR-EIL2, a transcriptional activator, is the transcriptional domain (Lee and Kim, 2003). In order to determine the transcriptional domain, PhEIL2 and its several deletion mutant vectors were constructed and the behavior of each construct was investigated in yeast cells. The full-length PhEIL2 protein fused to the GAL4 DNA-binding domain effectively activates the expression of the reporter gene of *lacZ* (**Figures 6A,B**), which indicates the function of PhEIL2 as a transcriptional activator in yeast. Further transcriptional activity analysis of various deletion mutants of PhEIL2 showed that the transcription-stimulating activity was still apparent when the C-terminal regions (353–470 and 471–612 amino acids) were fused to the GAL4 DNA-binding domain; both PhEIL2_353-470_ and PhEIL2_471-612_ mutant protein contained β-galactosidase activity while the PhEIL2_1-352_ mutant protein did not (**Figure 6**). Thus, the 353–612-amino acids at C-terminal of PhEIL2 play an important role for the function of PhEIL2 as a transcriptional activator.

### PhEIL2 Interacts with PhEIL1 by Y2H and BiFC Assays

**FIGURE 7:**
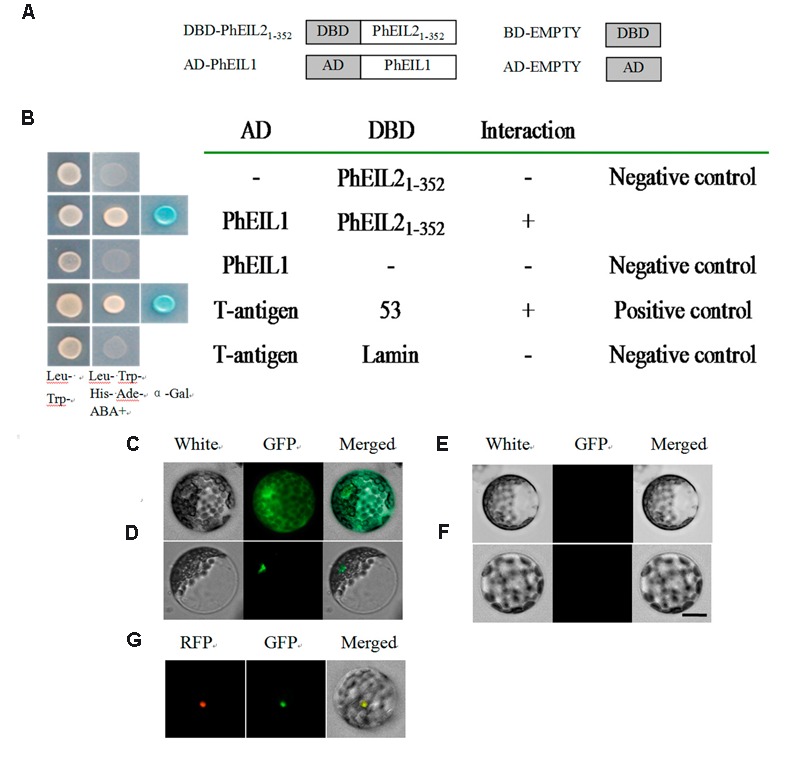
**Interaction between PhEIL2_1-352_ and portions of PhEIL1. (A,B)** Yeast two-hybrid assays between PhEIL2_1-352_ and portions of PhEIL1. **(A)** Portions of PhEIL2 and PhEIL1 were fused to DBD and AD, respectively; **(B)** interaction between PhEIL2_1-352_ and PhEIL1. Gold Y2H yeast strains were co-transformed with DBD–PhEIL2_1-352_ and PhEIL1. The ability of yeast cells to grow on synthetic medium lacking tryptophan, leucine, histidine, and adenine but containing 125 μM Aureobasidin A as well as turn blue in the presence of the chromagenic substrate X-β-Gal was scored as a positive interaction. Yeast cells transformed with pGBKT7-53+pGADT7-T, pGBKT7–PhEIL2_1-352_+pGADT7-T, pGBKT7–PhEIL2_1-352_+pGADT7-PhEIL1, pGBKT7+pGADT7-PhEIL1, or pGBKT7-Lamin+pGADT7-T were included as positive or negative controls. C-F, BiFC assays between PhEIL2 and PhEIL1. Translational fusion constructs of the coding region of PhEIL2 to pSAT-YFC and the coding region of PhEIL1 to pSAT-YFN were transferred into petunia protoplasts and tested for fluorescence complementation. **(C)** pSAT-GFP; **(D)** YFC-PhEIL2 and YFN-PhEIL1; **(E)** YFC-PhEIL2 and pSAT-YFN; **(F)** pSAT-YFC and YFN-PhEIL1. **(G)** YFC-PhEIL2, YFN-PhEIL1, and RFP-EOBII. Three biological replicates do these results represent in **(G)**. Images of **(C–F)** were captured with a confocal laser scanning system. Scale bars: 5 μm.

Since EILs could interact with its target as dimmers (Solano et al., 1998) and both PhEIL1 and PhEIL2 are involved in flower senescence in petunia, it is necessary to test whether PhEIL1 interacts with PhEIL2 in petunia and yeast two-hybrid (Y2H) assay was performed. To avoid this activation of the reporter gene, the 5′ ORF of *PhEIL2* cDNA sequence, encoding N-terminal half of the deduced protein (amino acids 1–352), and the full length cDNA of *PhEIL1* were subcloned into the pGBKT7 and pGADT7 vectors to form pGBKT7-PhEIL2_1-352_ bait vector and pGADT7-PhEIL1 prey vector, respectively. The derivative vectors of pGBKT7 and the pGADT7 were co-transformed into the yeast strain Y187. Yeast cells co-transformed with pGBKT7- PhEIL2_1-352_+pGADT7-PhEIL1 grew on selective medium lacking Trp, Leu, His, and Ade in the presence of 5 mM 3-AT. On the contrary, yeast cells harboring pGBKT7+pGADT7-PhEIL1, or pGADT7-PhEIL2_1-352_+pGBKT7 could not grow on the same selective medium (**Figures 7A,B**). These data showed that PhEIL2_1-352_ interacts with PhEIL1 in yeast.

To further test the interaction between PhEIL2 and PhEIL1, BiFC assay was performed. Co-expression of the C-terminal half of YFP fused to PhEIL1 (cYFP-PhEIL1) and the N-terminal half of YFP fused to PhEIL2 (nYFP-PhEIL2) in petunia protoplasts led to fluorescence with GFP as positive control. No interaction was detected between nYFP-PhEIL2 and cYFP, or between nYFP and cYFP-PhEIL1, which confirms the interaction between PhEIL2 and PhEIL1 (**Figures 7C–F**). In addition, previous study showed that EOBII was located in nuclear (Spitzer-Rimon et al., 2010) and with RFP (Red Fluorescent Protein)-EOBII as the nuclear maker, the interaction between PhEIL2 and PhEIL1 was further confirmed (**Figure 7G**).

## Discussion

Ethylene responsiveness in petunia corollas increases highly during flower senescence (Shibuya et al., 2004). In this study, a full-length cDNA of petunia *EIL* gene, *PhEIL2*, was isolated and the characters of *PhEIL2* and *PhEIL2* were identified during flower senescence.

When compared with other EILs described to date from different organisms, the N-terminal half of the deduced protein of PhEIL1 and PhEIL2 had high similarity to the corresponding regions of AtEIL3 and AtEIL1 in *Arabidopsis*, suggesting that they are indeed functional EIN3-like genes (Chao et al., 1997).

Previous research showed that other organisms *EILs* expression are regulated in tissue-specific manners (Waki et al., 2001; Lee and Kim, 2003; Iordachescu and Verlinden, 2005). Similarly, in this study, the relative expression pattern of the two different mRNAs varied in these tissues, with both *PhEIL1* and *PhEIL2* transcript being predominantly present in corollas and up-regulated during flower senescence.

The expression of most of *EILs* of other organisms, such as *Arabidopsis EIN3*, tomato *LeEIL*s, and mung bean *Vr-EIL1* and *Vr-EIL2*, was not significantly changed by the treatment of exogenous ethylene in grown plants (Chao et al., 1997; Lee and Kim, 2003). However, in this study, *PhEIL1* mRNA was up-regulated by ethylene, which is in line with the result of previous report (Shibuya et al., 2004), while *PhEIL2* transcriptional level is down-regulated by ethylene. Similarly, *DC-EIL3* mRNA showed significant accumulation while *DC-EIL1* mRNA showed significant reduction upon ethylene exposure in carnation (*Dianthus caryophyllus*) (Waki et al., 2001; Iordachescu and Verlinden, 2005). It is possible that the activities of EILs are controlled by a posttranslational mechanism (Chao et al., 1997; Kosugi and Ohashi, 2000; Tieman et al., 2001; Lee and Kim, 2003). Moreover, EIN3 and EIL1 proteins are degraded by EBF1/2 in ethylene signaling (Potuschak et al., 2003).

In petunia, pollination induced an ethylene burst and consequently floral senescence (Shibuya et al., 2004). The mRNA levels of both *PhEIL1* and *PhEIL2* were significantly increased after pollination 8 h by qPCR analysis, although *PhEIL2* expression showed down-regulated by exogenous ethylene treatment, suggesting that *PhEIL2* expression is regulated by multi-factors after pollination.

The etiolated T2 generation seedlings of *Arabidopsis* plants overexpressing the *EIN3* and *EIL1* genes and *TEIL* cDNA, showed a phenotype of constitutive triple response under the condition of lack of exogenous ethylene (Chao et al., 1997; Kosugi and Ohashi, 2000). Consistent with these results, VIGS-mediated both *PhEIL1* and *PhEIL2* silencing delayed flower senescence in petunia in this study. Furthermore, the expression of two senescence-associated genes, *PhERF3* and *PhCP2*, decreased in flowers in which VIGS-mediated silencing of *PhEIL1* and *PhEIL2* occurred. In addition, both *PhCHS/PhEIL1* and *PhCHS/PhEIL2* silencing led less ethylene production than *PhCHS* suppression in white flowers in days 4 and 5 after anthesis, which may suggest it exerts feedback control over ethylene production and the reduced ethylene evolution could be due to the partial block of ethylene autocatalysis. These results showed that both PhEIL1 and PhEIL2 are involved in flower senescence. On the other hand, in tomato, antisense plants with reduced transcription of a single *LeEIL* did not result in notable changes in ethylene response, but reduced the mRNA levels of multiple *LeEIL* reduced significantly ethylene response, showing functional redundancy of *LeEILs* in tomato (Tieman et al., 2001).

To uncover the transcriptional activation domain of PhEIL2, yeast one-hybrid assay was performed using several PhEIL2 deletion mutants. The results showed that the C-terminal 353-612 amino acid region that consists of 260 amino acid residues is the essential domain for transcription-stimulating activity. In contrast, the essential domain for transcription-stimulating activity in mung bean VR-EIL2 was laid in the acidic region that comprises 50 amino acid residues in N-terminal. In our experimental conditions, the PhEIL2_1-353_ mutant protein has only the background level of activity in yeast, suggesting that it is not essential domain for transcriptional activation. Further analysis showed that acid domain of PhEIL2 shares only 58.3% identity with that of Vr-EIL2. These results showed that essential domain for transcription-stimulating activity is not conserved in different EILs or in different species and requires further study.

Previous study suggested that homodimers of both EIN3 and EIL1 proteins are able to bind the promoters of *ERF1*, while EIN3 and EILs are not capable of forming heterodimers in *Arabidopsis* (Solano et al., 1998). In contrast, in this study, PhEIL2 interacts with PhEIL1 by Y2H and BiFC assays. In addition, both PhEIL1 and PhEIL2 are involved senescence. So, it is possible that heterodimers of PhEIL1 and PhEIL2 recognize their targets *in vivo*.

**FIGURE 8:**
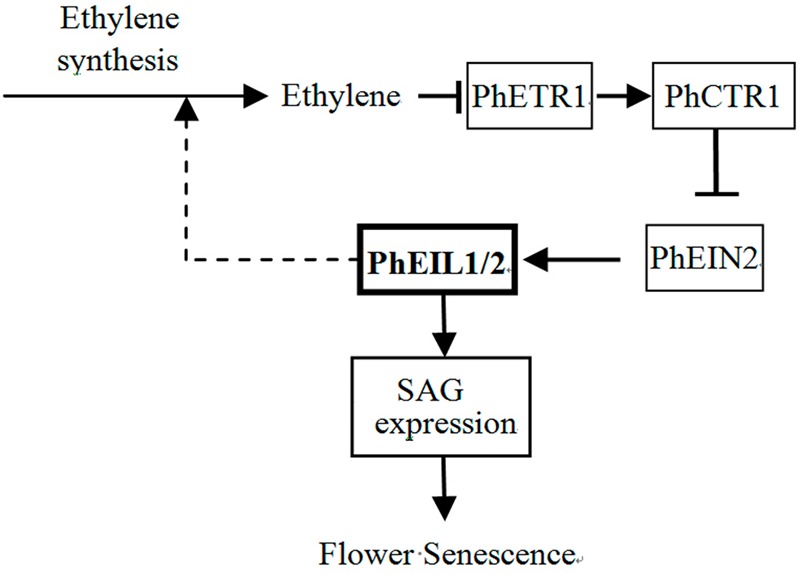
**Proposed models for the involvement of PhEIL1 and PhEIL2 in flower senescence in petunia**.

Based on the experimental data presented here and the model of ethylene signaling in *Arabidopsis* (Ji and Guo, 2013), we proposed a model to explain the involvement of the PhEIL1 and PhEIL2 in senescence in petunia flowers (**Figure 8**). In the present of ethylene, ethylene binds the receptor PhETR1 and ethylene signaling is transmitted to PhEIL1 and PhEIL2 heterodimers through PhCTR1 and PhEIN2 in petunia flowers (Shibuya et al., 2004). The heterodimers then activate the expression of senescence-related genes and accelerate senescence of flowers. In contrast, PhEIL1 or PhEIL2 suppression leads to the reduction of the heterodimers and the expression of senescence-related genes, and delays senescence of flowers. At the same time, ethylene production is reduced in petunia corollas, suggesting that PhEIL1 and PhEIL2 heterodimers could be involved in the regulation of the biosynthesis of ethylene in petunia flowers.

## Author Contributions

YY and JL designed research. FL, LH, YC, HL, and JL performed research. YY and FL, wrote paper.

## Conflict of Interest Statement

The authors declare that the research was conducted in the absence of any commercial or financial relationships that could be construed as a potential conflict of interest.

## Footnotes

1 http://www.ncbi.nlm.nih.gov

2 https://blast.ncbi.nlm.nih.gov/Blast.cgi
