# Supplementary material for: Molecular Characterization and Functional Analysis of Two Petunia PhEILs
Source: Front Plant Sci. 2016 Nov 1;7:1606. doi: 10.3389/fpls.2016.01606 (PMC5088505; doi:10.3389/fpls.2016.01606)
Supplement: Supplementary file 1 [file Data_Sheet_1.pdf]

Supplemental Table S1. Comparative analysis of PhEIL amino acid sequences with its closest homologs in *Arabidopsis*, tomato and *Vigna radiata*

Identity (%)	PhEIL1	PhEIL2	AtEIL1	AtEIL2	AtEIL3	AtEIN3	SlEIL1	SlEIL2
PhEIL2	35.1	100						
AtEIL1	35.6	62.7	100					
AtEIL2	29.7	40.2	41.1	100				
AtEIL3	50.1	39.3	39.3	32.5	100			
AtEIN3	34.6	61.8	76.2	41.8	39.6	100		
SlEIL1	35.6	86.2	61.5	40.9	38.7	62.4	100	
SlEIL2	34.3	74.8	61.9	42.0	37.8	62.7	74.9	100
VrEIL2	33.3	64.1	62.0	40.7	39.0	61.4	64.2	63.8

Supplemental Table 2 Primer sequences of *PhEIL1, PhEIL2, PhERF2, PhERF3, PhCP2* and *PhACTIN* used in quantitative real-time PCR

Gene	Forward primer (5′→3′)	Reverse primer (5′→3′)
PhEIL1	TTTCCATCTCCACTTACTATTG	ATATCAAGCCCCAAATTAAA
PhEIL2	CACTGGAAATCTACCTGG	TCCCCACTTGACTACTTG
PhERF2	TGTTGAAGATGCCCGTCCAG	TAGCTCGTTCACAGTGTTTCC
PhERF3	GCTTATGACGTTGAGGCA	CCTTCTGAGGATTTTGCT
PhCP2	AGCAAAGCAAACAAGGGAT	CAGCACCAACAATAAAGAAGC
PhACTIN	TGCTGATCGAATGAGCAAGGAA	GGAGCAACAACCTTAATCTTC

Supplemental Table 3 Primer sequences of *PhEIL1*, *PhEIL2* and *PhCHS* used in VIGS

Gene	Forward primer (5′→3′)	Reverse primer (5′→3′)
PhEIL1	TGTCATTCTTTGCCTCAT	CCGGAATTCTGGATTCCTGCCTTTACT
PhEIL2	CGCGGATCCCAAGTCACTCTATACTCCCACA	CCGGAATTCATCCACCTGACATAGCAGAC
PhCHS	GATCTCGAGTGGAGGCATTCCAACCATTG	CCAGAGCTCATTCAAGACCTTCACCAG

Supplemental Table 4 Primer sequences of *PhEIL2* used in activate transcription assay

Gene	Forward primer (5′→3′)	Reverse primer (5′→3′)
PhEIL2 (Full)	CCGGAATTCATGATGATGTTTGATGAAATGGGGT	CGCGGATCCGCTTAGAACCAAATTGGAGCA
PhEIL2 (1-352)	CCGGAATTCATGATGATGTTTGATGAAATGGGGT	CGCGGATCCTCAACAAAGTGAGATGGTCCG
PhEIL2 (353-612)	CCGGAATTCGAGTATGATGTTGAAGGTGCT	CGCGGATCCGCTTAGAACCAAATTGGAGCA
PhEIL2 (353-470)	CGGAATTCGAGTATGATGTTGAAGGTGCT	GCGGATCCACCTTGATTAACAGGCTGAGC
PhEIL2 (470-612)	CGGAATTCCCACCTTCCTTTGATCTATCT	GCGGATCCGCTTAGAACCAAATTGGAGCA

Supplemental Table 5 Primer sequences of *PhEIL1* and *PhEIL2* used in Y2H assay

Gene	Forward primer (5′→3′)	Reverse primer (5′→3′)
PhEIL1	CCGGAATTCTCAGTGATGAGGAGATTGAGCCA	CGCGGATCCCTATGAGGCAAAGAATGACATAA
PhEIL2(full)	CCGGAATTCATGATGATGTTTGATGAAATGGGGT	CGCGGATCCTCAACAAAGTGAGATGGTCCG
PhEIL2(1-352)	CCGGAATTCGAGTATGATGTTGAAGGTGCT	CGCGGATCCGCTTAGAACCAAATTGGAGCA

Supplemental Table 6 Primer sequences of *PhEIL1* and *PhEIL2* used in BiFC assay

Gene	Forward primer (5′→3′)	Reverse primer (5′→3′)
PhEIL1	CCGGAATTCAGTGATGAGGAGATTGAGCCA	CGCGGATCCCTATGAGGCAAAGAATGACATAA
PhEIL2	CCGGAATTCGATGATGATGTTTGATGAAATGGGGT	CGCGGATCCGCTTAGAACCAAATTGGAGCA

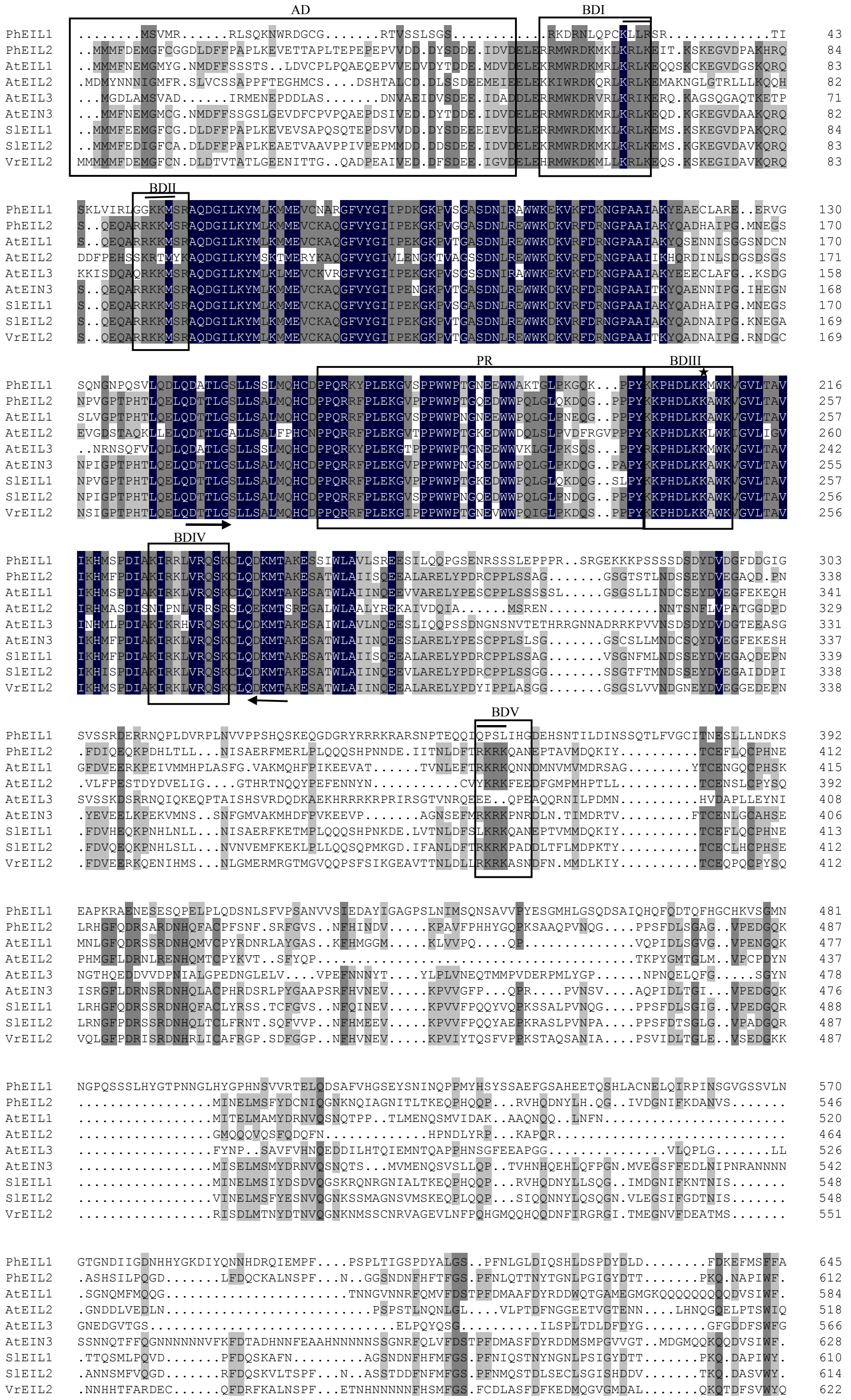

Supplemental Figure S1 Predicted amino acid sequence alignments of *PhEIL1* and *PhEIL2* with Arabidopsis AtEIL1 (AY065191), AtEIL2 (AY248904), AtEIL3 (AY248905) and AtEIN3 (NM_112968), and tomato SlEIL1 (AF328784), SlEIL2 (AF328785) and SlEIL3 (AF328786), respectively. The conserved amino acid residues are shaded in gray. The amino-terminal acidic domain

(AD), Pro-rich region (PR), and five small basic domains (BDI-V) are boxed. Asterisk refers to the Lys residue essential for the function of Arabidopsis EIN3. The putative nuclear-localized signals are indicated by solid lines. The arrows depict the primer amino acid sequences for reverse transcriptase-PCR. Dashes show gaps in the amino acid sequences introduced to optimize alignment.

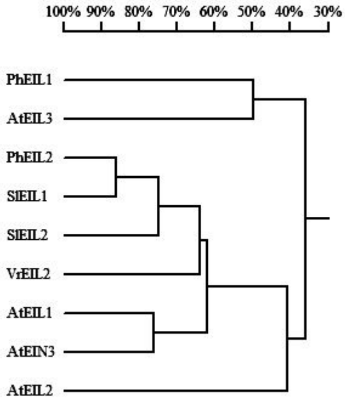

Supplemental Figure S2 Neighbor-joining trees among proteins encoded by EIL genes of petunia, Arabidopsis, mung bean and tomato using DNAMAN.
